# Rapid Assessment for Coexistence of Vitamin B12 and Iron Deficiency Anemia among Adolescent Males and Females in Northern Himalayan State of India

**DOI:** 10.1155/2013/959605

**Published:** 2013-07-22

**Authors:** Ashok Bhardwaj, Dinesh Kumar, Sunil Kumar Raina, Pardeep Bansal, Satya Bhushan, Vishav Chander

**Affiliations:** ^1^Department of Community Medicine, Dr. Rajendra Prasad Government Medical College, Kangra, Himachal Pradesh 176001, India; ^2^Department of Biochemistry, Dr. Rajendra Prasad Government Medical College, Kangra, Himachal Pradesh 176001, India

## Abstract

Coexistence of folic acid and vitamin B12 deficiency has been observed among adolescents with iron deficiency anemia, but limited evidence is available from India. So, a rapid assessment was done to study the prevalence of iron, folic acid, and vitamin B12 deficiency among adolescent males and females in northern Himalayan state in India. *Methods*. Total 885 (female: 60.9%) adolescents (11 to 19 completed years) were surveyed from 30-cluster village from two community development blocks of Himachal Pradesh. Serum ferritin, folic acid, and vitamin B12 were estimated among randomly selected 100 male and 100 female adolescents. *Results*. Under-nutrition (BMI < 18.5 kg/m^2^) was observed among 68.9% of adolescents (male: 67.1%; female: 70.7; *P* = 0.29). Anemia was observed to be prevalent among 87.2% males and 96.7% females (*P* = 0.00). Mild form of anemia was observed to be the most common (53.9%) form followed by moderate (29.7%) anemia. Strikingly, it was found that all the adolescents were deficient in vitamin B12 and none of the adolescents was observed to be deficient in folic acid. *Conclusion*. Among both male and female adolescents anemia with vitamin B12 deficiency was observed to be a significant public health problem. Folic acid deficiency was not observed as a problem among surveyed adolescents.

## 1. Introduction

Iron deficiency anemia is still a condition of a major public health concern for researchers and policy makers [1]. Period of adolescence is a significant phase of life as the physiologic growth spurt requires adequate nutrition in order to achieve healthy adulthood. Iron deficiency anemia reflects the state of undernutrition among adolescents. It results due to inadequate nutrition, blood loss, and inflammatory and infectious diseases. Iron deficiency anemia occurs because of poor intake and absorption is the most common form of anemia [2]. In India, the prevalence of iron deficiency anemia had been reported to be 55.8% among females and 30.2% among males in age group of 15–19 years [3]. Adolescent girls of 11–19 years across 16 districts observed the prevalence of anemia to be 90.0%, which was significantly more as compared to national level survey [4]. Iron requires haemglobin (Hb) synthesis in red blood cells and low level of Hb clinically determines anemia. In addition to iron, the haematopoiesis requires sufficient amount of other nutrients, like folic acid and vitamin B12 require red blood cells production [5]. 

Folic acid is a water soluble vitamin involved in nucleic acid, blood cell, and nervous tissue synthesis. It is widely distributed in green leafy food items “foliage” and its deficiency will lead to megaloblastic anemia due to prolongation of synthesis phase of red blood cells and retarded maturation of germ cells in bone marrow. In addition to folic acid, vitamin B12 deficiency is the second common cause for megaloblastic anemia. Vitamin B12 is required for two important transmethylation reaction, one of which closely associated with folate in DNA synthesis and haematopoiesis. Not plants, but nonvegetarian food items are the source of vitamin B12 [6]. Low level of vitamin B12 has been considered to affect reproduction and can cause recurrent abortion, infertility, and preterm abortion among pregnant mothers [7]. Limited studies reported that there is coexistence of folic acid and vitamin B12 deficiency along with iron deficiency anemia [8, 9]. This coexistence was also observed among adolescent girls [10–13]. 

Iron, folic acid, and vitamin B12 deficiency is expected among adolescents with poor nutrition. Their deficiency is of concern in India, as undernutrition was observed among about 60.0% of female and 45.0% male adolescents [3]. Also, surveys have also observed significant prevalence of iron deficiency anemia among adolescents [3, 4]. Understanding their possible interrelationship and global concern due to their deficiency and limited evidence from country, a rapid assessment was done to study the prevalence of iron, folic acid, and vitamin B12 deficiency among adolescent males and females in northern Himalayan state in India. Majority of study population of the study area is consuming mainly vegetarian diet. Cereals, pulses, and rice are consumed daily by almost all the families of studied adolescents (both males and females). 

## 2. Methods

It was a community based cross-sectional study done at Nichar block of district Kinnaur and Shahpur block of district Kangra, Himachal Pradesh, India. Nichar block has ranging altitude of 2,320 to 6,816 meters, and Shahpur has 427 to 6,401 meters above the sea level. A community-based survey was conducted in year 2010 by an independent trained field staff. During the survey total 885 adolescents (from 11 to 19 completed years) were surveyed and assessed for BMI and hemoglobin status. Serum ferritin, folates, and vitamin B12 were estimated among randomly selected 100 male and 100 female adolescents. Study participants were selected from study area using 30-cluster sampling technique, and the village was considered as a unit of cluster. From each cluster the estimated adolescents to be recruited were calculated by population proportion to size (PPS) method. Every cluster was hypothetically divided into four equal parts and study participants were recruited from each part for equal representation of cluster. Randomly, a house from each part was selected and adolescents were recruited till the sample size was met. Field staff administered a structured pretested interview-based questionnaire. 

After the interview anthropometric assessment (height and weight) was done, and 5 mL venous blood sample was collected. For Hb estimation, blood (20 *μ*L) was transferred to Whatman filter paper no. 1 and dried at room temperature. The paper was sealed in an envelope and transported to laboratory. The portion of filter paper with blood was placed in 5 mL Drabkin's solution and vortex for 5 minutes. After allowing solution to stand for 2 hours hemoglobin was assessed at 540 nm by spectrophotometer. For serum ferritin, folates, and vitamin B12 blood samples were centrifuged for separation of serum at the collection site and transported in cryocan (liquid nitrogen) to field laboratory for assessment. Ferritin, folates, and vitamin B12 were analyzed at IMMULITE 1000 (Chemiluminescent enzyme immunoassay) with control run. Kits of the same company (Siemens Health care diagnostics Products limited) were used while performing the tests [14]. 

Standard diagnostic criteria were used for low hemoglobin (male: Hb < 13 g/dL; female: <12 g/dL), low ferritin (<12 ng/mL), vitamin B12 (<200 pg/mL), and folates (<2.7 ng/mL) levels [15]. Degree of deficiency was assessed for anemia as severe (male: Hb < 9.0 g/dL; female: <7.0 g/dL), mild (male: Hb 9–11.9 g/dL; female: 7.0–10.9 g/dL), and moderate (male: Hb 12.0–12.9 g/dL; female: 11.0–11.9 g/dL). As used in National family Health Survey (NFHS-3), the Body Mass Index (BMI) was categorized as moderate/severe thin (BMI < 17.0 kg/m^2^), mild thin (BMI 17.0–18.4 kg/m^2^), normal (BMI < 18.4–24.9 kg/m^2^), overweight (BMI 25.0–29.9 kg/m^2^), and obese (BMI > 30.0 kg/m^2^) [3]. Ethical clearance was sought from institute ethics committee (IEC) before the data collection. Informed consent was also sought before interview and collection of blood sample. Statistical analysis was done by using Epiinfo 3.2.5 version (CDC), Chi square (*χ* ^2^), and unpaired student *t*-test was used to compare the proportions and means, respectively, [16]. As the study is rapid assessment that leads to differential distribution study participants subgroups (gender and age groups), the *P* value should be interpreted with caution.

## 3. Results

Total 885 (female: 60.9%) adolescents were surveyed. The mean age was found to be 14.0 (SD: ±2.5) and 14.8 (SD: ±2.5) year for males and females, respectively (*P* = 0.00). Participants were assessed for height and weight with an average BMI of 17.4 (SD: ±3.2) kg/m^2^ for males and 17.2 (SD: ±2.9) kg/m^2^ for females (*P* = 0.33). Assessment for anemia observed that the mean levels of Hb and ferritin among male (12.2 (SD: ±0.9) g/dL and 39.4 (SD: ±8.5) ng/mL) as compared to female adolescents (10.2 (SD: ±0.8) g/dL and 30.9 (SD: ±7.8) ng/mL) were significantly high (*P* = 0.00). On further assessment, it was found that among males the Hb level was 12.1 (SD: ±0.9) g/dL and 12.4 (SD: ±0.9) g/dL in age group of 11 to 15 and 16 to 19 years, respectively, and it was observed to be significant (*P* = 0.00). Whereas, in females the mean Hb was observed to be significantly (*P* = 0.00) low (9.9 (SD: ±0.8) g/dL) in age group of 16 to 19 years as compared to 11 to 15 years (10.9 (SD: ±0.9) g/dL). It was found that the average level of vitamin B12 (*P* = 0.05) and folate (*P* = 0.08) was low in age group of 16 to 19 as compared to 11 to 15 years. Among females of 16 to 19 year of age the average vitamin B12 was observed significantly higher (*P* = 0.03) (Table 1). 

The mean vitamin B12 level was observed to be 34.7 (SD: ±11.5) and 33.5 (SD: ±11.0) pg/mL among both male and female adolescents, respectively (*P* = 0.87), which were very much low as per the required level of 200 pg/mL. Whereas, average folates level was observed to be within normal limits for both male (15.2 (SD: ±6.5) ng/mL) and female (14.1 (SD: ±6.1) ng/mL) adolescents (*P* = 0.40). 

**Figure 1:**
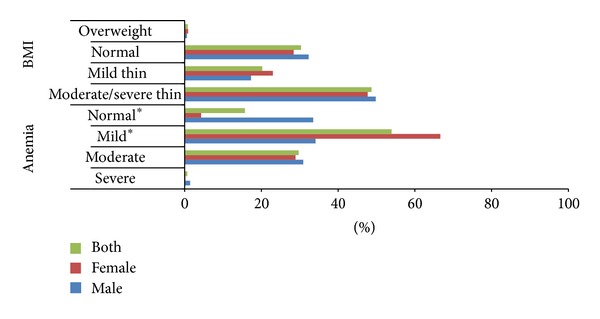
Prevalence of anemia and low BMI among studied adolescent males and females in Himalayan state of north India, 2010.

Undernutrition (BMI < 18.5 kg/m^2^) was observed among 68.9% of adolescents (male: 67.1%; female: 70.7; *P* = 0.29), and the prevalence of moderate/severe level of thinness (BMI < 17.0 kg/m^2^) was observed to be 49.8% and 47.7% among male and females, respectively (*P* = 0.63). Absolute 23.0% of female and 17.3% of male (*P* = 0.32) adolescent had mild level of thinness (BMI: 17.0–18.4 kg/m^2^). Based upon standard cutoffs anemia was observed to be prevalent among 87.2% males and 96.7% females (*P* = 0.00). Mild form of anemia was observed to be most common (53.9%) form followed by moderate (29.7%) anemia. Severe anemia was observed to be very less (0.7%) among studied adolescents. Significantly (*P* = 0.00) more of female (66.6%), as compared to male (34.1%) adolescents, were exposed to mild anemia, whereas the prevalence of moderate anemia was observed to be 30.9% among male and 28.9% among female adolescents (*P* = 0.72) (Figure 1). 

**Figure 2:**
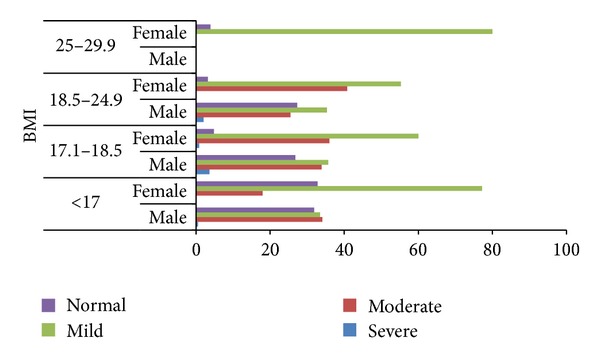
Prevalence of anemia as per different BMI categories among studied adolescents in Himalayan state of north India, 2010.

When assessed for prevalence of anemia among adolescents with their BMI, it was found that mild level of anemia was most common among all adolescents in all the categories of BMI. It was further observed that the prevalence of mild anemia was decreasing and moderate anemia was increasing among adolescents with moderate/severe thinness to normal appearance (Figure 2). Gender specific distribution of type of anemia to different categories of BMI showed that significantly more of females were exposed to mild form of anemia in all categories of BMI. For adolescents with BMI of less than 17.0 kg/m^2^, the prevalence of moderate anemia was 34.1% for males and 18.0% for females (*P* = 0.06) (Table 2). 

The prevalence of low ferritin was also observed to 15.0%, which was more among females (17.4%) than of males (12.2%) adolescents (*P* = 0.31). Strikingly, it was found that all the adolescents were deficient in vitamin B12 and none of adolescent was observed to be deficient in folic acid. 

## 4. Discussion

Among adolescents, anemia remains a significant public health problem which reflects nutritional deficiency. It affects negatively on physical growth, morbidity, cognition, and reproduction [17]. It was further observed that, in addition to iron, folic, and vitamin B12 deficiency also predisposes the adolescent girls to preterm, low birth weight, and other congenital malformations in newborn during their period of pregnancy [5]. Very less studies were carried out among adolescent males, and the present study observed high prevalence of low Hb (male: 87.2% and females: 96.7%) in both genders, which was found to be similar as reported to be 90.0% among adolescent girls [4], whereas country wide survey did report that 55.8% of female and 30.2% of male adolescents but of 15–19 years had anemia [3], which was observed to be low as compared to present and earlier study [4]. 

Present study had showed that the mild anemia was of most common type, whereas, very less adolescents were observed with severe anemia. Mild anemia was observed among 53.9% of adolescents, whereas, it was 48.4% in country wide survey [3]. However, present study observed low prevalence of severe anemia (0.7%) as reported as 1.6% in national survey [3]. Moderate anemia was observed to be common (60.1%) and severe anemia among 13.1% studied adolescents girls in survey conducted in selected districts. But survey of a different selected district (different of present study area) but of the same state in which present study was carried out the prevalence of mild, moderate, and severe anemia was observed as 31.0%, 28.0%, and 2.0%, respectively, [4]. As compared to national survey, present study and survey in 16 different districts have observed the high prevalence of anemia, which could be attributed to different Hb estimation method in country wide survey (Hemocue) and in other studies (indirect cyanmethemoglobin) [18]. As the serum ferritin level determines the level of iron stores in the body, present study had observed the prevalence of low serum ferritin to be 12.2% among male and 17.4% among females. It was observed to be low when compared with study, which had reported the prevalence of low serum ferritin as 27.3% among adolescent girls with anemia [19]. Iron stores are expected to be low among anemic adolescents but can be further attributed to different poor dietary consumption and absorption of iron rich food items. 

Folic acid and vitamin B12 deficiency impairs DNA and folate synthesis causing impaired and ineffective erythropoiesis [5]. Folic acid deficiency has been observed with iron deficiency, and the high prevalence was observed as 80.0% to 90.0% among studied adolescents in Venezuela [11, 12]. But about only 25.0% adolescent girls were observed to be deficient in folic acid in rural area of Bangladesh [13]. Strikingly, in the present study none of adolescent was observed to be deficient in folic acid and also the average level of folic acid was observed to be within normal limits. 

It has been discussed that the vitamin B12 deficiency may also coexist with iron deficiency, and so far, with the existing background of evidences, there is an insignificant contribution of vitamin B12 deficiency, burden of anemia in the world [20]. However, sprouting evidence has shown that now the prevalence of vitamin B12 deficiency is becoming a major type of nutritional deficient anemia. Its prevalence was observed to be around 20.0% among elderly population [12] and women of childbearing age [21]. A study among pregnant women of northern Indian state had revealed high prevalence (74.1%) of vitamin B12 deficiency [22]. Study among adolescents had shown that about 25.0% to 30.0% were deficient in vitamin B12 [11]. But the prevalence was only 7.0% for vitamin B12 deficiency that was observed among adolescent girls in rural area of Bangladesh [13]. A study found that that, among anemic adolescents, there was high prevalence of folic acid than that of vitamin B12 deficiency, whereas all the vitamin B12 deficient adolescents were anemic [12]. Present study showed that almost all of adolescents (about 90.0%) were anemic and all the studied adolescents were deficient in vitamin B12. 

With the limited available evidence among adolescents, in the present study, a consistent finding for prevalence and type of anemia, but strikingly inconsistent observation like zero prevalence of folic acid deficiency and 100.0% prevalence of vitamin B12 deficiency was observed. Variation across the geographical regions for nutrient deficiencies like iron, folates, and vitamin B12 is associated with nutrition profile and prevalence of various inflammatory and infectious diseases in adolescents. Present study observed high prevalence of undernutrition among adolescents. Moderate/severe thinness was observed to be more common followed by mild thinness. In country wide survey—among adolescent of 15–19 years of age—the comparable prevalence of moderate/severe (male: 58.1%; female: 46.8%) and mild level of thinness (male: 28.8%; female: 25.9%) was observed [3]. So, observed 70.0% undernutrition prevalence, it is attributed to less consumption of recommended calorie intake and deficiency of other micro-nutrients. But present study observed no deficiency in folic acid, which could be associated with consumption of diet rich in folic acid, the iron folic acid supplementation (national program) in the study area. However, detailed dietary assessment and consumption of IFA tablets were not done in the present study and therefore cannot be attributed. Apart from them, the method of analysis and determined levels for nutrients use in various studies could also explain the variation. 

Various surveys and studies across the country have shown anemia as an endemic public health problem. Country has initiated the distribution of IFA tablets among adolescents and pregnant women in order to reduce the anemia. In addition, various food supplementation programs for children, adolescents, and pregnant women have been in operation over the last three decades [23]. But, despite the significant efforts by the country the significant reduction in anemia is yet to be observed. A marked difference in IFA consumption due to considerable disparities in socioeconomic factors and health care delivery system was observed [24]. 

In present study, the iron deficiency anemia does not feel likely, as there was less prevalence of low serum ferritin and none of adolescent was observed to be deficient for folic acid. But inability to carry out the detailed hematological examination like total iron binding capacity, transferring receptor and peripheral blood smear should be considered as limitations of the study. Another limitation of study is that the estimation of Hb using dried filter paper could possibly lead to underestimation of Hb but could not be accounted as it was not validated. But considering the high prevalence of low hemoglobin and vitamin B12 deficiency among adolescents in present study would further require a carefully planned analytical study in order to establish the association of vitamin B12 deficiency and anemia. So, in addition to IFA supplementation, the role of other multivitamins in reducing anemia is becoming a major area of research. 

## Figures and Tables

**Table 1:** Age and gender distribution of average Hb, ferritin, vitamin B12, and folate levels among studied adolescent males and females in Himalayan state of north India, 2010.

Variables Mean (±SD)	Gender	Age group (years)		*t*, *P* value
		11–15	16–19	
Hb	Male	12.1 (0.9)	12.4 (0.9)	3.3, 0.00
	Female	10.4 (0.8)	9.9 (0.8)	6.0, 0.00
	All	11.3 (1.2)	10.9 (1.4)	4.6, 0.00

Ferritin	Male	39.1 (11.2)	40.3 (11.5)	0.2, 0.83
	Female	27.5 (12.0)	34.3 (10.4)	1.9, 0.05
	All	39.4 (12.5)	36.0 (12.0)	0.8, 0.42

Vitamin B12	Male	36.0 (10.2)	30.3 (6.9)	0.4, 0.65
	Female	14.6 (7.5)	23.3 (3.5)	2.1, 0.03
	All	39.4 (21.0)	25.1 (4.2)	1.9, 0.05

Folate	Male	16.0 (6.4)	12.1 (3.7)	1.3, 0.18
	Female	14.6 (4.5)	13.5 (3.1)	0.9, 0.34
	All	15.0 (5.3)	13.1 (3.3)	1.7, 0.08

**Table 2:** Prevalence of anemia (g/dL) as per different BMI (kg/m^2^) categories among studied adolescent males and females in Himalayan state of north India, 2010.

BMI	Severe		*χ* ^2^, *P* value	Moderate		*χ* ^2^, *P* value	Mild		*χ* ^2^, *P* value
	Male	Female		Male	Female		Male	Female	
	<9.0	<7.0		9.0–11.9	7.0–9.9		12.–12.9	10.–11.9	
<17.0	1 (0.5)	0 (0.0)	—	62 (34.1)	45 (18.0)	3.4, 0.06	61 (33.5)	193 (77.2)	41.0, 0.00
17.1–18.5	2 (3.6)	1 (0.8)	—	19 (33.9)	45 (36.0)	0.8, 0.35	20 (35.7)	75 (60.0)	3.9, 0.04
18.5–24.9	2 (2.0)	0 (0.0)	—	26 (25.5)	62 (40.8)	1.4, 0.23	36 (35.3)	84 (55.3)	3.5, 0.06
25.0–29.9	0 (0.0)	0 (0.0)	—	0 (0.0)	9 (20.0)	—	(0.0)	4 (80.0)	—

—: could not be calculated due less numbers.
